# Leukemia cutis in a patient with acute monocytic leukemia diagnosed simultaneously with hepatocellular carcinoma: A case study

**DOI:** 10.3892/ol.2013.1580

**Published:** 2013-09-12

**Authors:** DONG KEUN SEOK, SAE YOON KEE, SOON YOUNG KO, JUNG HWA LEE, HYE YOUNG KIM, IN SUN KIM, HEE YEON SEO

**Affiliations:** 1 Department of Internal Medicine, Konkuk University School of Medicine, Chungju-si, Chungcheongbuk-do 380-701, Republic of Korea; 2 Department of Pediatrics, College of Medicine, Korea University, Seongbuk-gu, Seoul 136-703, Republic of Korea; 3 Department of Anesthesia and Pain Medicine, College of Medicine, Korea University, Seongbuk-gu, Seoul 136-703, Republic of Korea; 4 Department of Pathology, College of Medicine, Korea University, Seongbuk-gu, Seoul 136-703, Republic of Korea

**Keywords:** synchronous primary neoplasms, hematologic neoplasm, solid neoplasm

## Abstract

Acute myeloid leukemia presenting as leukemia cutis (LC) with hepatocellular carcinoma is extremely rare. The current study presents a case of a 53-year-old male with generalized cutaneous nodules on the face and anterior chest wall. Laboratory tests, including bone marrow biopsy revealed acute myelomonocytic leukemia (AML-M4) with skin and tonsilar involvement. Liver magnetic resonance imaging (MRI) revealed a 6-cm mass in hepatic segments 4 and 8, and a liver biopsy demonstrated that hepatocellular carcinoma cells and immature blast cells coexisted. Although LC has been reported in Korea, a case of LC associated with acute myelomonocytic leukemia was diagnosed simultaneously with hepatocellular carcinoma and tonsillar involvement. The present study describes this case with a review of the literature.

## Introduction

Leukemia cutis (LC) is a rare condition that is characterized by the presence of leukemic cell infiltration into the dermis, subcutis, skin adnexa and blood vessels (1). The lesions usually present with subcutaneous nodules, multiple papules or violaceous or brown plaques (2). LC has various cutaneous manifestations, which may make it difficult to distinguish the disease from other dermatoses. The incidence of LC was reported in 2–3% of patients with acute myeloid leukemia (AML) (1).

Liver cancer is the fifth most common type of cancer in Korea (3). Hepatocellular carcinoma (HCC) accounts for 85–90% of all primary liver cancers, with a median survival of less than one year (4). The most common risk factors for HCC are chronic infection with hepatitis B virus or hepatitis C virus. Heavy alcohol intake is a well-established HCC risk factor and aflatoxin, obesity and diabetes are also possible risk factors (5,6).

Multiple primary cancer is defined as a specific malignant tumor, manifesting as more than one primary tumor that is diagnosed in the same patient, either simultaneously or sequentially (7,8). Synchronous multiple primary cancer is defined as two or more tumors occurring within six months of each other. The incidence of synchronous multiple primary cancer is uncommon and it is even less common to observe a synchronous solid tumor with a hematological malignancy. Recently, cases of solid tumor synchronously presenting with hematological malignancy were reported (7). However, the global synchronous occurrence of two primary malignancies presenting as AML and hepatocellular carcinoma is rare, particularly in cases of AML presenting as LC with HCC. Although LC has been reported in Korea, a case of LC associated with AML that was diagnosed simultaneously with hepatocellular carcinoma and tonsillar involvement has been identified in the present study. Therefore, the present study describes this case with a review of the literature. Written informed consent was obtained from the patient’s family.

## Case report

**Figure 1:**
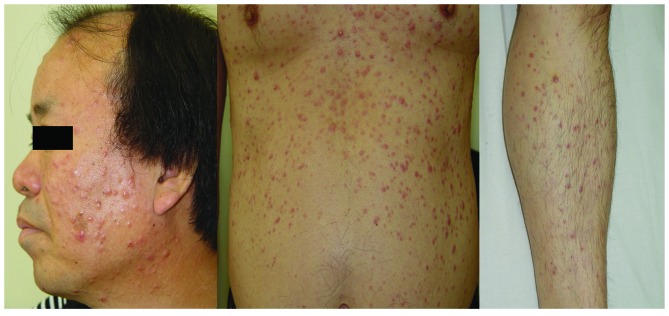
Generalized cutaneous nodules on the face and anterior chest wall.

**Figure 2:**
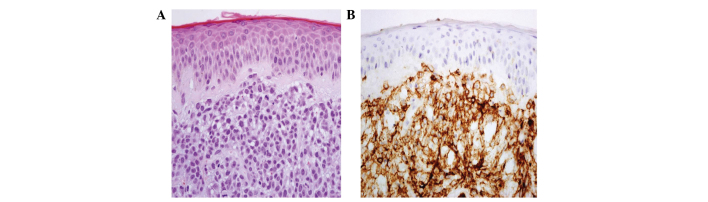
Skin biopsy. (A) Atypical monomorphic tumor cells with round to oval vesicular nuclei and a moderate amount of cytoplasm infiltrating the upper and deep dermis and the periappendiceal tissue (H&E staining; magnification, ×400. (B) Tumor cells are positive for leukocyte common antigen and CD43, but negative for CD34, CD68, C-kit, cytokeratin, myeloperoxidase and S-100 protein (immunohistochemical staining; magnification, ×400). H&E, hematoxylin and eosin.

**Figure 3:**
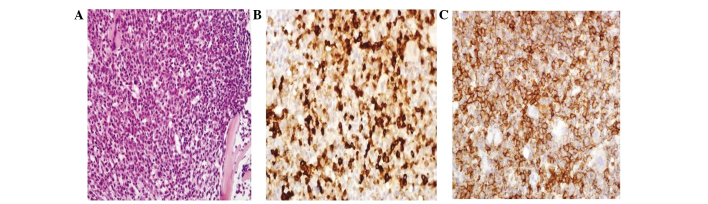
Bone marrow biopsy. (A) Bone marrow biopsy showing packed marrow with increased atypical cells with large nuclei, irregular nuclear membranes, dispersed chromatin and nucleoli and relatively abundant cytoplasm (H&E staining; magnification, ×400). (B–D) The cells are positive for (B) myeloperoxidase, (C) CD43 and (D) CD68 (immunohistochemical staining; magnification, ×400). HE, hematoxylin and eosin.

**Figure 4:**
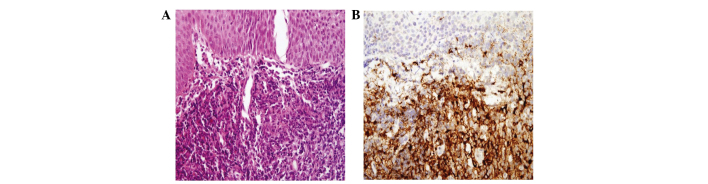
Right tonsillar punch biopsy. (A) Biopsied specimen consists of several irregular fragments of tonsils, showing diffuse infiltration of atypical cells. (H&E staining; magnification, ×400). (B) Infiltrated cells are positive for myeloperoxidase, but negative for CD34, CD3 and CD20 (immunohistochemical staining; magnification, ×400). H&E, hematoxylin and eosin.

**Figure 5:**
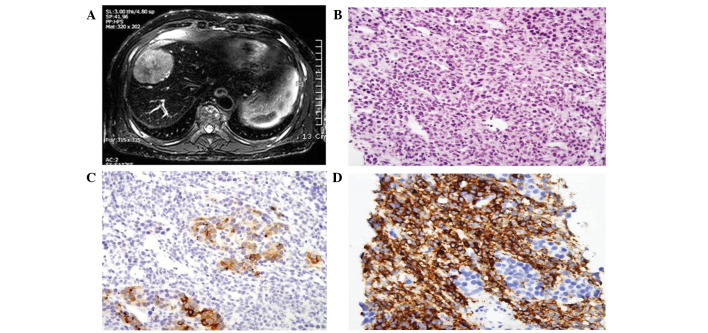
Liver (A) MRI and (B–D) biopsy. (A) A 6-cm ill-defined mass in hepatic segments 4 and 8. (B) Hepatocellular carcinoma cells and immature blast cells were observed (H&E staining; magnification, ×400). (C) Liver biopsy specimen show focal positivity for AFP (immunohistochemical stain; magnification, ×400). (D) Immature blasts infiltrate the hepatocellular carcinoma. These cells are CD43-positive, but negative for myeloperoxidase and CD34 (immunohistochemical stain; magnification, ×400). MRI, magnetic resonance imaging; H&E, hematoxylin and eosin; AFP, α-fetoprotein; HCC, hepatocellular carcinoma.

A 53-year-old male was referred to Konkuk University Chungju Hospital (Chungju, Korea) for generalized cutaneous nodules on the face, anterior chest wall and legs (Fig. 1), as well as a sore throat. The patient had no history of medical systemic disease, with the exception of hypertension. Furthermore, there was no history of fever, trauma, weight loss, bleeding or any systemic illness. However, the patient was a chronic alcoholic. At admission, the hemoglobin level was 7.0 g/dl, the platelet count was 10.3×10^3^/μl and the white blood cell (WBC) count was 20.8×10^3^/μl. A differential count revealed the following: Neutrophils, 10%; monocytes, 25%; blast cells, 24%; carcinoembryonic antigen, 1.0 ng/ml; carbohydrate antigen 19-9, 12.2 U/ml; α-fetoprotein (AFP), 1,650 ng/ml; erythrocyte sedimentation rate, 99 mm/h; and C-reactive protein, 14.1 mg/l. The serology studies, which included hepatitis B surface antigen, antibodies to hepatitis C virus and a serum antibody titer against HIV, *Mycobacterium* and *Cytomegalovirus*, were all negative. Hematoxylin and eosin (H&E) staining of the upper and deep dermis and a periappendiceal tissue skin biopsy specimen demonstrated atypical monomorphic tumor cells with round to oval vesicular nuclei and a moderate amount of cytoplasm infiltration (Fig. 2). In addition, the immunohistochemical staining revealed that the tumor cells were positive for leukocyte common antigen and CD43, but negative for CD34, CD68, C-kit, cytokeratin, myeloperoxidase and S-100 protein. H&E staining of the bone marrow biopsy revealed packed marrow with increased atypical cells, large nuclei with nuclear membranes, dispersed chromatin and abundant cytoplasm. Furthermore, the cells were positive for myeloperoxidase, CD43 and CD68 (Fig. 3). H&E staining of the right tonsillar punch biopsy demonstrated several irregular fragments of tonsils that revealed diffuse infiltration of the atypical cells. The immunohistochemical staining revealed that the cells were positive for myeloperoxidase, but negative for CD34, CD43 and CD20 (Fig. 4). Liver magnetic resonance imaging (MRI) demonstrated a 6-cm ill-defined mass in hepatic segments 4 and 8. H&E staining of the liver biopsy revealed immature blasts infiltrating the hepatocellular carcinoma that were positive for CD3 and CD68, but negative for myeloperoxidase and CD34 (Fig. 5).

A diagnosis of acute myelomonocytic leukemia (AML-M4) was confirmed with skin, tonsil and hepatic involvement and HCC. The patient was administered standard induction chemotherapy with idarubicin (12 mg/m^2^/IV for 1–3 days) and cytarabine (100 mg/m^2^/IV for 1–7 days). On the 36th day, the bone marrow examination demonstrated complete remission with 2.2% blast cells. With regard to the HCC, the patient was treated by transcatheter hepatic arterial chemoembolization (TACE). The patient was scheduled to undergo consolidation chemotherapy, but the complete blood count revealed a WBC count of 21.7×10^3^/μl (72% blasts). Therefore, relapse of AML was suspected. The patient did not wish to undergo further treatment and succumbed shortly thereafter, due to the progression of AML.

## Discussion

The prevalence of multiple synchronous primary neoplasm is 2–10% in all the carcinomas that have been reported in literature (8). The patients who are diagnosed with cancer may have a 20% higher risk of a new primary cancer compared with the general population (8). The synchronous occurrence of two primary malignancies presenting as AML and hepatocellular carcinoma is rare. LC refers to neoplastic leukemic cells that infiltrate into the skin, most often in conjunction with systemic leukemia. In the biopsy-comfirmed LC case studies, the prevalence of this disease was 2–3%. Skin involvement in myeloid leukemia is associated with monocytic differentiation, central nervous system involvement and aneuploidy of chromosome 8 (9). Furthermore, AML accompanied by LC has an aggressive and poor clinical course (10,11). In the present study, liver MRI was performed due to the history of chronic heavy alcoholism and an elevated AFP level of 1650 ng/ml was observed. The MRI revealed a 6-cm ill-defined mass in hepatic segments 4 and 8. The liver biopsy revealed that HCC cells and immature blast cells coexisted. Following a diagnosis of acute myelomonocystic leukemia, standard induction chemotherapy with idarubicin and cytarabine was administered. Furthermore, the 6-cm HCC lesion, the patient’s general condition and the laboratory results made it impossible to perform a partial hepatectomy. The 6-cm mass was too large for radiofrequency ablation and percutaneous ethanol injection. Therefore, TACE was performed. However, prior to the consolidation chemotherapy, relapse of AML was suspected, but the patient refused further treatment for AML. As described previously, LC may be an indicator of acute leukemia with poor prognosis. The patients who are diagnosed with a primary cancer may develop a second primary cancer with variable clinical manifestations. Due to the lack of guidelines for the standard management of such a condition, the treatment modality should be determined through therapeutic strategies that are specific to the disease and performance status of the patient.
